# Association of Parkinson’s Disease With Microbes and Microbiological Therapy

**DOI:** 10.3389/fcimb.2021.619354

**Published:** 2021-03-08

**Authors:** Zhao-Ji Chen, Cheng-Yu Liang, Li-Qing Yang, Si-Min Ren, Yan-Min Xia, Lei Cui, Xiao-Fang Li, Bu-Lang Gao

**Affiliations:** Department of Neurology, Affiliated Hospital of Hebei University, Baoding, China

**Keywords:** Parkinson’s disease, a-synuclein, microbe, microbiological therapy, pathology

## Abstract

Parkinson’s disease (PD) is the most common movement disorder in the world, affecting 1–2 per 1,000 of the population. The main pathological changes of PD are damage of dopaminergic neurons in substantia nigra of the central nervous system and formation of Lewy bodies. These pathological changes also occur in the intestinal tract and are strongly associated with changes in intestinal flora. By reviewing the research progress in PD and its association with intestinal flora in recent years, this review expounded the mechanism of action between intestinal flora and PD as well as the transmission mode of α - synuclein in neurons. In clinical studies, β diversity of intestinal flora in PD patients was found to change significantly, with *Lactobacillusaceae* and Verrucomicrobiaceae being significantly increased and Lachnospiraceae and Prevotellaceae being significantly decreased. In addition, a longer PD course was associated with fewer bacteria and probiotics producing short chain fatty acids, but more pathogenic bacteria. Moreover, the motor symptoms of PD patients may be related to Enterobacteriaceae and bacteria. Most importantly, catechol-O-methyltransferase inhibitors and anticholinergic drugs could change the intestinal flora of PD patients and increase the harmful flora, whereas other anti-PD drugs such as levodopa, dopamine agonist, monoamine oxidase inhibitors, and amantadine did not have these effects. Probiotics, prebiotics, and synbiotics treatment had some potential values in improving the constipation of PD patients, promoting the growth of probiotics, and improving the level of intestinal inflammation. At present, there were only a few case studies and small sample studies which have found certain clinical efficacy of fecal microbiome transplants. Further studies are necessary to elaborate the relationship of PD with microbes.

## Introduction

As the most common movement disorder in the world, Parkinson’s disease (PD) affects 1–2 per 1,000 of the population (Nair et al., 2018), and about 7–10 million people suffer from PD today (Tysnes and Storstein, 2017). The incidence of this disease is expected to double in the next twenty years in proportion to the increase of aging population (Calabrese, 2007). Besides the primary symptoms of motion (akinesia, tremor, rigidity, and postural instability), many non-motor symptoms, including anosmia, orthostatic hypotension, constipation, fatigue, depression, pain, and anhedonia, may even take place before the motor signs and accomplish the clinical spectrum (Dehay and Fernagut, 2016). The main pathological changes of PD are damage of dopaminergic neurons in substantia nigra of the central nervous system and formation of Lewy body (LB) which is also called Lewy pathology (LP). In PD, conformational transformation refolds native α-helical α-synuclein (α-SYN) into pathology-associated β-sheet α-SYN to efficiently form the LB (Ghosh et al., 2017) and further deposit in the brain. It can induce degeneration and loss of dopaminergic cells in the substantia nigra compacta, eventually leading to motor symptoms (Ubeda-Bañon et al., 2014). It has been confirmed that LP occurs not only in the central nervous system (CNS), but also in peripheral tissues (Malek et al., 2014), such as the gut (Pfeiffer, 2020). Moreover, the pathological changes of LP in the peripheral nervous system also affect the function of the normal enteric nervous system (ENS), even before the CNS is affected by the pathological changes (Bridi and Hirth, 2018). Current clinical evidence suggests that gastrointestinal symptoms and olfactory dysfunction occur before motor symptoms in the early stage of PD (Ross et al., 2008; Cersosimo et al., 2013). It has been shown that chronic constipation symptoms in PD patients are positively correlated with the pathological severity of LP (Lebouvier et al., 2010). The intestinal tract and the CNS in PD patients have the same LP basis, and before the onset of PD, gastrointestinal dysfunction has been presented significantly, which shows that the gut and PD are closely related. In recent years, the hypothesis that PD originates from the intestine has attracted more and more attention, and we reviewed the current literature for possible association of PD with microbes and microbial therapy for PD.

## Mechanism of Action Between Intestinal Flora and PD

It was initially estimated that the number of microbial cells is 10 times that of human cells (Sender et al., 2016). Among them, bacteria account for the vast majority, mainly including Bacteroides and Firmicutes, as well as a small number of Proteobacteria, Actinobacteria, Fusobacteria, and Verrucomicrobia phyla (Grenham et al., 2011). In addition, there are bacteria, archaea, fungi, protozoa, viruses, and their collective genome found on and within the human body, which together constitute the microbial community (Barko et al., 2018). In mammals, the microbiome makes vital contributions to energy homeostasis, metabolism, gut epithelial health, immunologic activity, and neurodevelopment. Such a large microbial community has become an integral part of the host body. In terms of cell composition, genetic diversity and metabolic capacity, the host animal is a multi-species hybrid organism in the state of dynamic symbiosis between host and microbial cells (Voth and Khanna, 2020).

**Figure 1:**
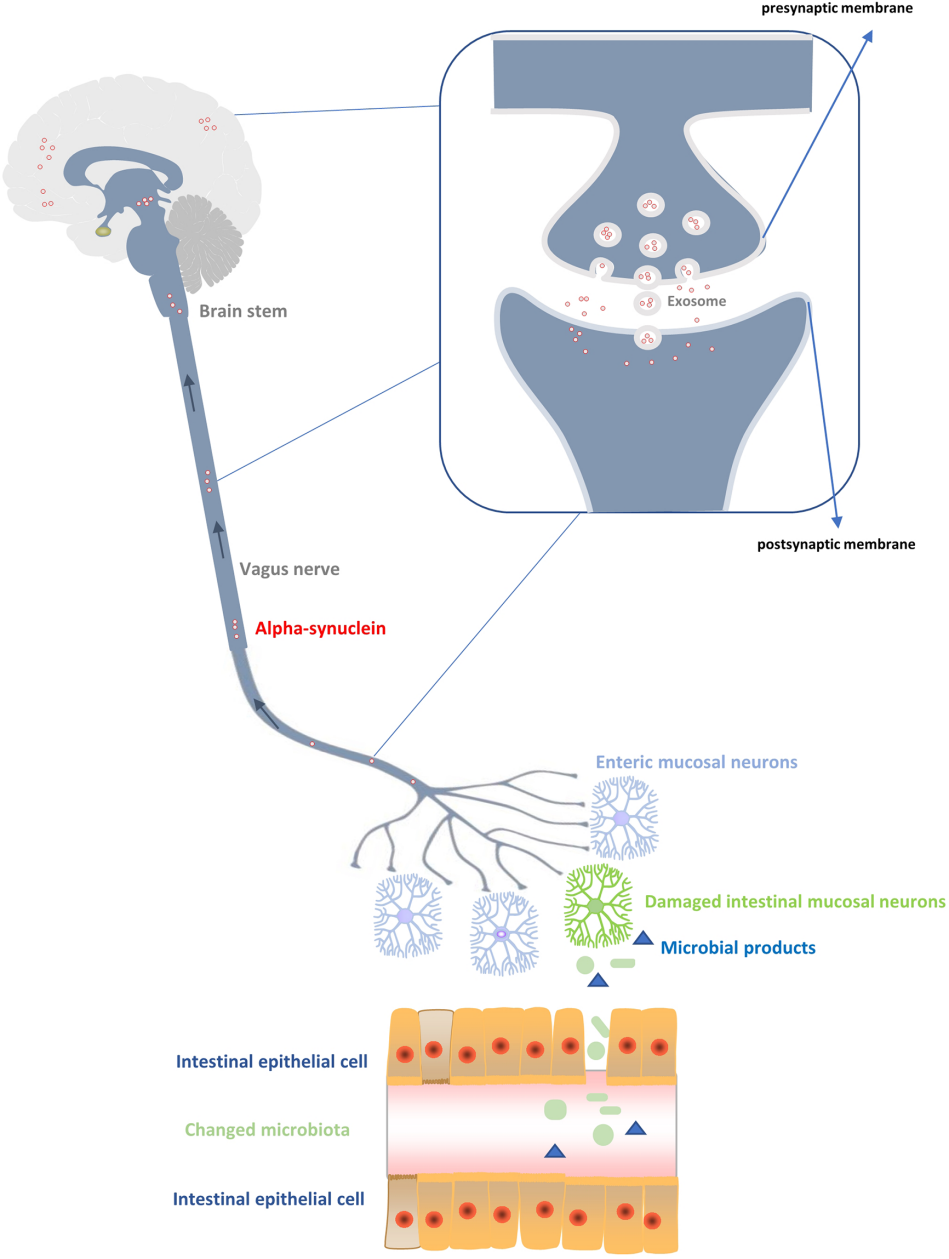
Production and transmission of α-synuclein to the central nervous system. Changes of the intestinal flora produce abnormal products with toxic effects on the peripheral intestinal ganglion, resulting in excessive production of α-synuclein. The α-synuclein is transmitted up the vagus nerve, medulla oblongata, and brain stem to reach the cortex, thus affecting the damage of neurons in the central system. During the transmission process, the main diffusion modes of α - synuclein between neurons are vesicular exocytosis and micropytosis in addition to the exosome transmission mode.

In 1980, researchers found the pathological structure of LP in the gastrointestinal tract of PD patients for the first time and proposed that PD might originate from the gastrointestinal tract (Edwards et al., 1992). On the basis of previous studies, Barrak et al. (2003a; 2003b; Hawkes et al., 2007) made some further exploration and put forward an important hypothesis on the pathogenesis of PD: PD may be caused by a neurotropic pathogen originating from the intestine (Hawkes et al., 2007). This pathogen induces oxidative stresses by initiating inflammatory response and then produces misfolded α-SYN (Braak et al., 2003a; Braak et al., 2003b), which spreads later to the brain in a way of “dominos” transmission (**Figure 1**). Microbiome is the main component of human intestinal tract, and it is the intermediary between human body and the environment. However, it is currently believed that many confounding factors play a pathogenic role in the pathogenesis of PD, including disorder of the brain-gut axis, neuroendocrine mechanism, inflammation, immunity, and endotoxin (Haikal et al., 2019; Yang et al., 2019; Kaur et al., 2020). A combination of the enterobacteria and their products rather than a particular enterobacteria seem to be responsible for the specific folding of α-SYN (Stolzenberg et al., 2017; Manfredsson et al., 2018; Terada et al., 2018). In a recent study, mice exposed to lipopolysaccharide (LPS) positive bacteria after intracerebral injection of α -SYN produced a unique fibrillary form of α-SYN (Kim et al., 2016). Therefore, bacterial contact, especially LPS positive bacteria, may be the driving force of α -SYN disease. In addition, another PD mouse model confirmed that LPS can produce a strong pro-inflammatory response *in vivo*, including strong glial activation and increases in TNF - α, IL-1 β, IL-6, and IL-10, besides 34% loss of dopamine neurons in the substantia nigra (Beier et al., 2017). Jangula et al. in 2013 showed no significant increase in the blood-brain-barrier permeability in α-SYN knockout mice exposed to LPS, however, the blood-brain barrier permeability of normal wild-type mice was significantly increased after LPS injection, with significantly increased expression of inflammatory factors in both groups of mice (Jangula and Murphy, 2013). This also shows the synergism of inflammatory factors and α - syn, which can be induced by LPS. Pietrucci et al. also demonstrated in 2019 that the pathways involved in the synthesis of LPS in PD were significantly more than those in healthy people (Pietrucci et al., 2019). In addition, the products of intestinal bacteria, such as short chain fatty acids (SCFAs), can affect blood gut permeability and blood brain barrier (Manfredsson et al., 2018). The destruction of these barriers and the subsequent translocation of bacterial metabolites to the brain have been considered as one of the causes of brain neuroinflammation (Perez-Pardo et al., 2017a). Current studies have also shown that SCFAs can cause dyskinesia and lead to α -SYN responsive microglia in the brain (Sampson et al., 2016). Evidence from Cirstea’s clinical study also suggests that PD patients have fewer bacterial clusters associated with SCFAs (Cirstea et al., 2020). Currently, the specific role of intestinal bacteria and α -SYN in the etiology of PD is not clear, but the exploration and understanding of the inflammatory process caused by intestinal flora may provide relevant clues for clinical treatment and the pathogenesis of PD.

### Transmission Mechanism of α - SYN

Through the study of yeast cell models, some scholars put forward for the first time the evidence of α-SYN secretion mechanism (Dixon et al., 2005) (**Figure 1**). It is believed that α -SYN enters the plasma membrane mainly through the endoplasmic Golgi secretion pathway, including transport by Golgi and secretory vesicles, connection and fusion of vesicles with plasma membrane before entering the synaptic space (Dixon et al., 2005). Although it has been shown that vesicular exocytosis is the most likely mechanism of α -SYN release (Emmanouilidou and Vekrellis, 2016), the result was not confirmed by another study on SHSY5Y cells and MES cells (Lee et al., 2005), which suggested that intracellular overload of α -SYN may lead to different intracellular localization and utilization of various cell release pathways (Emmanouilidou and Vekrellis, 2016). When α -SYN is excreted by exocytosis, exosomes play a key role. Neuronal exosomes are produced by fusion or detachment of neuronal membranes from synaptic polyvesicles with a size of about 30–50 nm (Johnson et al., 2020). Their main function is to promote intercellular communication by transporting specific proteins (such as heat shock proteins) or RNA (such as mRNA and miRNA) (Lancaster and Febbraio, 2005; Valadi et al., 2007; Gibbings et al., 2009). At present, studies have confirmed that α -SYN oligomers can utilize exosomes in neurons, and host cells can excrete α -SYN oligomers through exosomes. α -SYN in exosomes is more easily absorbed by endocytosis than α -SYN fibers (Danzer et al., 2012). In addition, calcium ions are also involved in this exocytosis process. The solubility of calcium ions is high, and its efflux effect is more obvious (Emmanouilidou et al., 2010). For transmission of α -SYN between neurons, it is necessary to have the uptake mode of cells besides the related secretion mechanism of neurons. There seems to be no natural barrier for the uptake of α -SYN by neurons in human body. Some studies have found that neurons in the body will absorb α -SYN within a few minutes after exposure to exogenous proteins (Luk et al., 2012). However, there is no clear evidence on how neurons uptake α -SYN. Two main uptake methods have been proposed, i.e., pinocytosis (Kisos et al., 2012; Karpowicz et al., 2017) and receptor-mediated endocytosis (Holmes et al., 2013; Mao et al., 2016). When neurotoxic α-SYN is uptaken, it will be ingested by lysosomes (Karpowicz et al., 2017). Lysosome integrity plays an important role in determining the pathological transmission rate of α -SYN between cells (Jiang et al., 2017). When lysosome breaks down, the released α -SYN aggregates again to form the pathogenic LP structure (Flavin et al., 2017) and transports to the distal synaptic terminals through microtubule axons (Holmqvist et al., 2014), thus completing the transmission process of α -SYN. In short, it is generally accepted that α -SYN can be transmitted between neurons, but there is no clear evidence for the specific transmission mechanism. Further studies are still needed to explore the internal relationship for complete understanding of the molecular mechanism of α -SYN transmission.

## Clinical Study of Intestinal Flora in Patients With PD

At present, there are 17 studies on the comparison between PD patients and healthy people (Keshavarzian et al., 2015; Scheperjans et al., 2015; Bedarf et al., 2017; Hill-Burns et al., 2017; Hopfner et al., 2017; Li et al., 2017; Petrov et al., 2017; Heintz-Buschart et al., 2018; Lin et al., 2018; Qian et al., 2018; Aho et al., 2019; Barichella et al., 2019; Pietrucci et al., 2019; Cirstea et al., 2020; Cosma-Grigorov et al., 2020; Qian et al., 2020; Zhang et al., 2020), including five studies from China (Li et al., 2017; Lin et al., 2018; Qian et al., 2018; Qian et al., 2020; Zhang et al., 2020), three from Germany (Bedarf et al., 2017; Hopfner et al., 2017; Cosma-Grigorov et al., 2020), two from Italy (Barichella et al., 2019; Pietrucci et al., 2019), two from Finland (Scheperjans et al., 2015; Aho et al., 2019), two from the United States (Keshavarzian et al., 2015; Hill-Burns et al., 2017), one from Luxembourg (Heintz-Buschart et al., 2018), one from Russia (Petrov et al., 2017), and one from Canada (Cirstea et al., 2020). A total of 13 studies reported the diversity of intestinal microflora, and the diversity index (α and β diversity) was primarily used to describe the overall characteristics of microbial community composition (**Table 2**) (Cosma-Grigorov et al., 2020). Among the 13 studies, α diversity was used as an index to measure the number of bacterial groups in a single fecal sample. Six of the 13 studies reported no significant (P>0.05) difference in α diversity between PD patients and healthy controls (**Table 2**). In the other seven studies, one study showed that the intestinal flora richness of PD patients was significantly decreased (Keshavarzian et al., 2015), and five studies revealed that the intestinal flora richness of PD patients was significantly increased (Li et al., 2017; Qian et al., 2018; Barichella et al., 2019; Qian et al., 2020; Zhang et al., 2020). Beta diversity emphasizes the similarity of intestinal microflora structure of different persons (**Table 2**). According to the current research data, the beta diversity of PD patients is significantly different from that of healthy people, and in longitudinal follow-up, the difference of beta diversity of intestinal microflora of PD patients becomes more obvious (Barichella et al., 2019; Cosma-Grigorov et al., 2020). The diversity of intestinal microorganisms in human body reflects the regulation of metabolism and immune system. Young people usually have a stable diversity of intestinal bacteria. With increase of age, the diversity of intestinal bacteria in human body gradually decreases, and the corresponding immune and metabolic regulatory functions are also gradually weakened, which provides the opportunity of invasion by opportunistic pathogens and intestinal inflammation (Vemuri et al., 2018).

Fifteen families and 11 genera of microbes had been reported (Keshavarzian et al., 2015; Scheperjans et al., 2015; Bedarf et al., 2017; Hill-Burns et al., 2017; Hopfner et al., 2017; Li et al., 2017; Petrov et al., 2017; Heintz-Buschart et al., 2018; Lin et al., 2018; Qian et al., 2018; Aho et al., 2019; Barichella et al., 2019; Pietrucci et al., 2019; Cirstea et al., 2020; Cosma-Grigorov et al., 2020; Qian et al., 2020; Zhang et al., 2020), of which 11 families and seven genera had been reported to increase in PD patients, while four families and four genera had been reported to decrease (see **Tables 1** and **2** for details). It is worth noting that the abundance of *Lactobacillusaceae* and *Verrucomicrobiaceae* have been reported to increase in PD patients for many times (six times and eight times, respectively), whereas at the generic level, the main increase is *akkermansia* (six times reported). In addition, the declines of Lachnospiraceae and Prevotellaceae in PD patients have been reported most frequently (eight times and seven times, respectively), whereas the main declines at the genus level are *prevotella*, *roseburia*, and *faecalibacterium*. Moreover, Lachnospiraceae has also been reported to increase abundantly in PD patients. In human body, Verrucomicrobiaceae is correlated with the expression of proinflammatory cytokines in plasma (Lin et al., 2019). *Lactobacillusaceae* may affect the activity of intestinal nervous system through vagus nerve afferent, besides affecting α -SYN and brain chemicals secreted by intestinal nervous system (Kunze et al., 2009). Increase of *Lactobacillusaceae* may regulate the function of dopamine in substantia nigra by affecting the secretion of ghrelin, thus limiting the degeneration of neurons (Scheperjans et al., 2015). Prevotellaceae and Lachnospiraceae are producers of SCFA, which can maintain the integrity and stability of intestinal barrier and promote gastrointestinal peristalsis (Canani et al., 2011). It can be seen that the effect of Enterobacteriaceae on PD is not a single inflammatory regulation, but a combination of mechanisms such as hormone regulation and neural regulation.

**Table 1:** Specific gut microbiota reported in 17 clinical studies.

Family	Genus
**Increased in PD**	
Lactobacillaceae (Holmqvist et al., 2014; Keshavarzian et al., 2015; Mao et al., 2016; Manfredsson et al., 2018; Lin et al., 2018; Haikal et al., 2019) (6/17)	Lactobacillus (Flavin et al., 2017; Hopfner et al., 2017; Lin et al., 2018; Haikal et al., 2019) (4/17)
Enterobacteriaceae (Keshavarzian et al., 2015; Mao et al., 2016; Manfredsson et al., 2018; Haikal et al., 2019) (4/17)	
Verrucomicrobiaceae (Jangula and Murphy, 2013; Holmqvist et al., 2014; Keshavarzian et al., 2015; Jiang et al., 2017; Hopfner et al., 2017; Lin et al., 2018; Aho et al., 2019; Zhang et al., 2020) (8/17)	Akkermansia (Jangula and Murphy, 2013; Keshavarzian et al., 2015; Jiang et al., 2017; Hopfner et al., 2017; Lin et al., 2018; Barichella et al., 2019; Aho et al., 2019; Zhang et al., 2020) (8/17)
Enterococcaceae (Holmqvist et al., 2014; Hopfner et al., 2017) (2/17)	
Bacteroidaceae (Jiang et al., 2017; Qian et al., 2018) (2/17)	Bacteroides (Jiang et al., 2017) (1/17)
Rikenellaceae (Li et al., 2017; Haikal et al., 2019) (2/17)	
Streptococcaceae (Scheperjans et al., 2015; Qian et al., 2018; Haikal et al., 2019) (3/17)	Streptococcus (1/17)
Clostridiaceae (Jiang et al., 2017) (1/17)	ClostridiumIV (Petrov et al., 2017) (1/17) ClostridiumXVIII (Petrov et al., 2017) (1/17)
Lachnospiraceae (Jiang et al., 2017; Petrov et al., 2017) (2/17)	
Bifidobacteriaceae (Jangula and Murphy, 2013; Scheperjans et al., 2015; Keshavarzian et al., 2015; Lin et al., 2018) (4/17)	Bifidobacterium (Jangula and Murphy, 2013; Flavin et al., 2017; Lin et al., 2018) (3/17)
Ruminococcaceae 57 (1/17)	
**Decreased in PD**	
Prevotellaceae (Holmqvist et al., 2014; Mao et al., 2016; Flavin et al., 2017; Li et al., 2017; Lin et al., 2018; Aho et al., 2019; Zhang et al., 2020) (7/17)	Prevotella (Holmqvist et al., 2014; Flavin et al., 2017; Hopfner et al., 2017; Li et al., 2017; Lin et al., 2018; Zhang et al., 2020) (6/17)
Lachnospiraceae (Jangula and Murphy, 2013; Scheperjans et al., 2015; Keshavarzian et al., 2015; Li et al., 2017; Manfredsson et al., 2018; Qian et al., 2018; Lin et al., 2018) (7/17)	Roseburia (Jangula and Murphy, 2013; Keshavarzian et al., 2015; Li et al., 2017; Lin et al., 2018) (4/17)
Ruminococcaceae (Jangula and Murphy, 2013; Holmqvist et al., 2014; Li et al., 2017; Lin et al., 2018) (4/17)	Faecalibacterium (Jangula and Murphy, 2013; Flavin et al., 2017; Li et al., 2017; Qian et al., 2018; Lin et al., 2018) (5/17)
Coprobacillaceae (Jiang et al., 2017) (1/17)	Coprococcus (Jiang et al., 2017; Lin et al., 2018) (2/17)

PD, Parkinson’s disease. The superscript number indicates the references. Numbers in the parenthesis indicates presence in the number of clinical studies.

**Table 2:** Summary of diversity of gut microbiota in PD patients in various studies.

Study	Year	Country	α Diversity indices	β Diversity indices
Scheperjans et al. (2015)	2015	Finland	P>0.05	P<0.02
Keshavarzian et al. (2015)	2015	USA	P>0.05	P<0.05
Petrov et al. (2017)	2017	Russia	p=0.001	p=0.0001
Hopfner et al. (2017)	2017	Germany	P>0.05	p = 0.0491
Li et al. (2017)	2017	China	P = 0.74	P < 0.001
Barichella et al. (2019)	2018	Italy	P=0.012	P=0.002
Qian et al. (2018)	2018	China	P<0.05	P<0.05
Lin et al. (2018)	2018	China	P>0.05	P>0.05
Pietrucci et al. (2019)	2019	Italy	p=0.1725	
Aho et al. (2019)	2019	Finland	P>0·1	P<0·017
Cirstea et al. (2020)	2020	Canada	P>0.05	P<0.001
Zhang et al. (2020)	2020	China	P<0.05	P = 0.001
Qian et al. (2020)	2020	China	P=0.0083	P=0.001
Cosma-Grigorov et al. (2020)	2020	Germany	P>0.05	P <0.047

PD, Parkinson’s disease.

PD is a clinical process of chronic degeneration of the nervous system, which has varied degrees of clinical characteristics in different clinical stages (van Rooden et al., 2011). Minato et al. in 2017 found that the total number of representative fecal bacteria in PD patients decreased during 2-year follow-up, and the decrease of *bifidobacteria* in the early stage can predict deterioration of PD (Minato et al., 2017). Different from the short-term follow-up result by Minato et al, the study by *baricella* et al. included PD patients with different courses of disease (Barichella et al., 2019). Longitudinal comparison demonstrated that with increase of the course of disease, there was an obvious increase of *lactobacillus* but a decrease of Lachnospiraceae in the intestinal tract of PD patients (Barichella et al., 2019). Keshavarzian et al. also found that the longer the course of PD, the fewer the anti-inflammatory bacteria such as Lachnospiraceae (Keshavarzian et al., 2015). In the study by Pietrucci et al. in 2019, the clinical stage of PD patients is correlated negatively with Lachnospiraceae but positively with Enterobacteriaceae, with the correlation being consistent with the sports injury (UPDRSIII scale). Although there are different reports about the course of disease and the composition of intestinal bacteria, it can be seen that the number of SCFA-producing bacteria and probiotics is decreased while the number of pathogenic bacteria is increased. At present, there are also views that α -SYN is bidirectional in the brain gut axis (Ulusoy et al., 2017). α -SYN can be transferred from the central nervous system to the intestine (Lebouvier et al., 2010), leading to dysfunction of intestinal ganglion and change of intestinal components. Therefore, it is not clear whether the change of intestinal bacteria is the result or cause of PD progression.

Motor symptoms are the most prominent manifestation of PD patients. Common motor symptoms include tremor, bradykinesia, postural disorders, and gait instability (Lebouvier et al., 2010). The severity of motor symptoms in PD patients is often related to the severity of the disease. For the first time, the Finnish team suggested that the increase in the abundance of Enterobacteriaceae in PD patients is associated with the non-tremor phenotype (postural disorders and gait instability) (Scheperjans et al., 2015). This argument has also been confirmed by Aho et al. (2019). However, researchers from China showed that the relative abundance of *Clostridium*, *verrucomicrobia*, and *akkermansia* in patients with tremor phenotype was significantly higher than that in patients with non-tremor subtype, and the increased abundance of *bacteroides* was positively correlated with the severity of motor symptoms (assessed by UPDRS scores) (Lin et al., 2018). An Italian study revealed that the degree of exercise damage in PD patients was positively correlated with the increase of *lactobacillus* but decrease of Lachnospiraceae (Barichella et al., 2019). Enterobacteriaceae and *bacteroidia* are believed to increase the endotoxin in the body of PD patients, promote release of inflammatory cytokines in the body, and, with blood circulation through the blood-brain barrier, further induce nerve inflammation and nerve death (Choi et al., 2016; Caputi and Giron, 2018).

In the treatment of PD, catechol-O-methyltransferase (COMT) inhibitors (Kaakkola, 2000) and anticholinergic drugs (Ness et al., 2006) are commonly used even though they may often cause gastrointestinal side effects. Scheperjans (Scheperjans et al., 2015)et al reported for the first time that the application of COMT inhibitors was positively correlated with the abundance of Enterobacteriaceae in PD patients. Hill burns et al. found that COMT inhibitors or anticholinergic drugs can reduce the abundance of *bifidobacterium* in PD patients (Hill-Burns et al., 2017). In contrast, Barichella et al. (2019) found that COMT inhibitors promoted increase of Lactobacilluslacteae but decrease of Lachnospiraceae in PD patients. In general, the gastrointestinal reactions of PD drugs are reflected in the changes of intestinal flora, resulting in primarily increase of pathogenic bacteria but decrease of anti-inflammatory bacteria. In a study investigating the functional implications of microbial and viral gut metagenome changes in PD patients in the early stage without the use of L-dopa (Bedarf et al., 2017), it was found that various combinations of anti-PD drugs (mainly dopamine agonists, monoamine oxidase inhibitors, and amantadine) had no significant effect on the intestinal bacteria in PD patients. Heintz bushart et al. analyzed PD patients taking levodopa and found that levodopa was an important covariate affecting the abundance of intestinal flora and duration of disease, but without affecting intestinal Enterobacteriaceae (Cosma-Grigorov et al., 2020). In order to evaluate the effect of antiparkinsonian drugs on microbial composition, Zhang et al. analyzed the relationship between four antiparkinsonian drugs (levodopa, dopamine agonist, anticholinergic drug, and amantadine) and the intestinal microflora of PD patients. Their results showed that none of the four antiparkinsonian drugs caused significant changes in the intestinal microflora of PD patients (Zhang et al., 2020). However, it is worth noting that a publication bias may exist in this study by Zhang et al. because 85% of the patients were treated with levodopa and only 8% were treated with anticholinergic drugs.

Clinical studies have been increasingly performed on Enterobacteriaceae in PD patients, however, due to the heterogeneity between these studies, it is impossible to make a direct comparison between them. Our review included 17 clinical studies investigating intestinal flora in PD patients and healthy controls from different countries, and the intestinal microbiome with geographic characteristics may affect phenotype-related microbiome changes among populations with different geographic backgrounds (Gaulke and Sharpton, 2018; He et al., 2018). Even if matched healthy spouses (Boertien et al., 2019) were included to eliminate the interference of diet and living habits and influence of age, gender, drugs, and other confounding factors on intestinal flora including the amount of exercise and coffee intake (Cosma-Grigorov et al., 2020), different courses and clinical severity of PD included in different studies may be the source of statistical differences and biases (Scheperjans, 2016; Aho et al., 2019; Ji et al., 2020) to say nothing of the significant differences in the intestinal bacteria structure among PD patients (Flores et al., 2014). Most importantly, the differences in specimen collection, delivery, primers, experimental procedures, and diet among different studies inevitably affect the accuracy of the results, which makes it difficult to reach a unified conclusion or to find a common microbial marker of PD (Boertien et al., 2019).

## Microbial Therapy

At present, there is no cure for Parkinson’s disease, and the treatment is primary through levodopa drugs to relieve symptoms. Unfortunately, levodopa treatment did not prevent the development of the disease (Schrag and Quinn, 2000), whereas many non-motor symptoms did not respond to dopaminergic treatment (Lee and Koh, 2015). With the understanding of the relationship between intestinal microflora and PD in recent years, researchers are trying to treat PD by adjusting the structure of intestinal microflora. Malnutrition occurs when the central reciprocity between microbiota members, metabolites, and the host immune system in a microbial ecosystem is lost. Generally, in a malnourished ecosystem, potentially pathogenic microorganisms take over at the cost of potentially beneficial microorganisms. Therefore, the strategy of transforming dysfunctional intestinal microbiota into health-related intestinal microbiota is the main principle of current intestinal microbiotherapy. The means to achieve it include use of probiotics, diet, and fecal microbiome transplantation (FMT) (Parashar and Udayabanu, 2017; Bailey and Holscher, 2018; Barko et al., 2018). These interventions may provide opportunities to supplement traditional PD treatment.

### Probiotics, Prebiotics, Synbiotics

There are many ways of dietary therapy for PD, such as a single component diet (vitamins, fatty acids, and minerals) and a variety of dietary components to complement each other (such as the Mediterranean diet). At present, there are a lot of clinical studies to explore the benefits and clinical value of the dietary therapy (Boulos et al., 2019). This review studied the relationship between microorganisms and PD and mainly focused on the effects of oral probiotics and prebiotics on PD. The concept of probiotics has been around for 17 years, and “living microorganisms, when applied in an appropriate amount, will bring health benefits to the host” (Gazerani, 2019). Therefore, the current requirements related to probiotics therapy should be met: resistance to gastric acid, resistance to bile and pancreatin, adhesion and colonization to intestinal mucosal cells. In addition, they can effectively colonize the host, resist antimicrobial substances produced by pathogenic bacteria, and have no translocation (Uyar and Yildiran, 2019). Currently, probiotics are mainly provided in the form of drugs, food, supplements, and formulas. The most commonly used probiotics (Azad et al., 2018) include several kinds of lactic acid bacteria, bifidobacteria, yeast, and other Escherichia coli. In a randomized, double-blind, placebo-controlled clinical trial in Iran, participants were randomly assigned to receive probiotics daily, including Lactobacillus acidophilus, Bifidobacterium bifidum, Lactobacillus reuteri, and Lactobacillus fermentum, or placebo (n = 25 per group, one capsule per day) for 12 weeks. Probiotics supplementation for 12 weeks significantly improved the gene expression of IL-1, IL-8, TNF - α, TGF – β, and PPAR - γ in PD patients, but did not affect the gene expression of VEGF and LDLR or the biomarkers of inflammation and oxidative stress (Borzabadi et al., 2018). In another randomized, double-blind, placebo-controlled clinical trial in Iran, 60 PD patients were treated with probiotics supplementation (probiotics: Lactobacillus acidophilus, Bifidobacterium composition, encapsulated Lactobacillus reuteri, and Lactobacillus fermentum) for 12 weeks to evaluate the effects on exercise and metabolic parameters. Compared with placebo, probiotics reduced the Movement Disorders Society-Unified Parkinson’s Disease Rating Scale scores, high-sensitivity C-reactive protein, malondialdehyde and insulin levels, insulin resistance, enhanced glutathione levels, and insulin sensitivity (Tamtaji et al., 2019). Baricella et al. studied the role of probiotics and prebiotic fibers in PD related constipation. In this study, 120 patients were randomly assigned (2:1) to placebo (n=40) or fermented milk (n=80) containing prebiotic fiber and probiotics including Thermophiles, Enterococcus faecium, Lactobacillus rhamnosus GG, Lactobacillus acidophilus, Lactobacillus plantarum, Lactobacillus paracasei, Lactobacillus delbrueckii subsp. Bulgaria and bifidobacteria once a day for 4 weeks. Compared with the placebo group, the probiotics group had more complete defecation (P = 0.002) (Barichella et al., 2016). Although current clinical trials have demonstrated the potential therapeutic value of probiotics, the precise mechanism of probiotics’ effect on PD remains to be elucidated, and the effect is likely to be produced through a variety of mechanisms (Fang, 2019). In addition, the clinical value of probiotics needs to be further explored because of the limitations of studies (Uyar and Yildiran, 2019).

Prebiotics are generally considered an inanimate food ingredient to give health benefits related to host microbial regulation and a substrate selectively utilized by the host microorganisms for health benefits, including non-food elements. As a promising tool in promoting the general health, preventing and treating numerous juvenile diseases, prebiotics are considered an agent of immunoaction, with the effects lasting beyond the active administration of the prebiotic. Because of its low risk of adverse effects, ease of application, and strong capability to influence the composition and function of intestinal microbiota, the beneficial clinical applications of prebiotics are expanding (Cencic and Chingwaru, 2010). Ideal prebiotics should be resistant to gastric acid, intestinal bile salts, and other hydrolases, nonabsorble by the upper digestive tract, and easy to be fermented by beneficial intestinal flora (Pandey et al., 2015). Prebiotics mainly include some non-digestible carbohydrates and oligosaccharides, such as fructooligosaccharides, galactooligosaccharides, inulin (which can also increase the absorption of calcium), lactulose (a synthetic disaccharide used as a drug for treating constipation and hepatic encephalopathy), lactulose, and lactulooligosaccharides (Pandey et al., 2015). Oligosaccharides, the most widely used oligosaccharides, can pass through the stomach and further reach the ascending colon, where they will be selectively metabolized by the inherent probiotic components of the microbiota, resulting in a significant decrease in pH and creating an unfavorable habitat for the growth of clostridia (Gagliardi et al., 2018). At present, there is no clinical study on the relationship between prebiotics and PD, but the relevant evidence for immune function, intestinal motility, and constipation reveals its potential clinical value of application (Perez-Pardo et al., 2017b).

Synbiotics refers to products containing both probiotics and prebiotics (Frei et al., 2015). Current research suggest that synbiotics can reduce the inflammatory indexes of healthy subjects, and the effect is better when combined with prebiotic fructooligosaccharides (FOS) (Rajkumar et al., 2015). In addition, a randomized, placebo-controlled trial found that dietary synbiotic supplementation improved the constipation symptoms (Ding et al., 2016), which was also observed in the above-mentioned experiment by Barichella et al. (2016). Nearly a quarter of PD patients sufferred from small intestines bacterial overgrowth (SIBO), especially in patients with advanced PD, who had a much higher incidence of SIBO than that of healthy people (Vizcarra et al., 2018). The effect of SIBO in PD patients is mainly related to the deterioration of gastrointestinal symptoms and motor function. The mechanism may include intestinal mucosal damage leading to ineffective drug absorption, and malabsorbed amino acids and bacterial degradation products competing with levodopa for saturated active transport system in the small intestine (Vizcarra et al., 2018). The study of Khalighi et al. showed that in SIBO patients, when the synbiotics containing Bacillus coagulans and prebiotics were added after antibiotic treatment, the treatment response was better than that of antibiotics alone. Combination therapy can also significantly reduce abdominal distension and diarrhea (Khalighi et al., 2014). So far, the research results of prebiotics/probiotics have changed a lot, reflecting the diversity of probiotic strains tested and the diversity of the population studied. However, the current studies generally pay much attention to the clinical value of probiotics, prebiotics, synbiotics, and other products for PD patients, but pay less attention to high osmotic pressure, gastrointestinal and abdominal distension, gastrointestinal discomfort, and other related side effects (Yoo and Kim, 2016). For patients with severe constipation, targeted probiotics may be beneficial. However, further studies are needed in more populations to determine their effectiveness, treatment modalities, and treatment duration. Large scale studies, especially well-designed randomized controlled trials, are essential to prove the safety and effectiveness of these supplements.

### Fecal Microbiome Transplantation (FMT)

FMT is considered to be a more comprehensive method to restore the intestinal microbiota. Its main purpose is to re-establish an intestinal microbiota through sending the feces of healthy donors (the whole intestinal microbiota) into the digestive tract of patients (Gazerani, 2019). It has been used in the treatment of gastrointestinal infections or other diseases, and has been approved by the World Health Organization and the USA Federal Drug Administration (FDA) for clinical treatment (Carlucci et al., 2016). FMT includes screening, homogenization, filtration, and reuse of fecal samples for specific microbiota, followed by colon administration or oral administration in the form of oral tube or capsule containing lyophilized substances (Liu et al., 2020). Currently, it is believed that direct administration of FMT by colonoscopy is the most effective approach, but attention should be paid to the risk of bleeding. For patients with intestinal obstruction or severe colitis, the effective rate of FMT through administration of nasointestinal tube or esophagogastroduodenoscopy can still reach 81% to 86% (Kim and Gluck, 2019). Capsule delivery is the latest form of FMT. Capsule seems to be a reasonable choice for patients with contraindications of colonoscopy. The advantages of capsule delivery are to reduce the operation time, cost and complications of colonoscopy and to eliminate the need of colon preparation. However, at present, the efficiency of capsule delivery needs to be further evaluated (Kim and Gluck, 2019).

At present, the mechanism of FMT in the treatment of PD is not clear. A mouse experiment showed that fecal microbiota transplantation (FMT) reduced the imbalance of intestinal microbiota, reduced fecal SCFA, alleviated body damage, and increased the content of Da and 5-HT in striatum of PD mice (Sun et al., 2018). Furthermore, FMT reduced the activation of microglia and astrocytes in substantia nigra, and decreased the expression of TLR4/TNF - α signaling pathway components in intestine and brain, thereby reducing the level of inflammation *in vivo (* Sun et al., 2018). These factors have been confirmed to be related to the pathogenesis of PD. Nonetheless, few large-scale studies have been performed on the treatment of PD and FMT. A case report from China demonstrated that FMT significantly reduced the short-term (one week) tremor of lower limbs and constipation in patients with PD, whereas the facial and movement symptoms have not been significantly improved (Huang et al., 2019). Moreover, the α diversity of intestinal flora in PD patients was similar to that in healthy donors, and the abundance of lachnoccitridium, ruminococcaceae, akkermansia, and faecalibacterium was increased (Huang et al., 2019). Another study from China with 15 PD patients demonstrated significantly (P<0.05) improved in the sleep state (PSQI score), quality of life (PDQ-39), anxiety and depression (HAMD score and HAMA score), and motor symptoms at one and three months of follow-up (Xue et al., 2020). In this study (Xue et al., 2020), ten patients received fecal microbiota suspension *via* colonoscopy and five patients received fecal microbiota suspension *via* nasointestinal route, and the colonic route had been revealed to have a better and longer curative effect than the nasointestinal route (Xue et al., 2020). However, the application of FMT in the treatment of PD is still at the initial stage. Although the outcomes of treatment are positive, some issues still need to be addressed in the use of FMT, including ethical issues, selection of appropriate donors, risk and benefit assessment, impact on patients’ mental state and behavior, and long-term safety (Feng et al., 2018). It has been suspected that FMT only replaced the lumen microbiome rather than the mucosal microbiome (Van Laar et al., 2019). Most importantly, current clinical studies on FMT in the treatment of PD demonstrated that FMT did not have a stable long-term curative effect (Huang et al., 2019; Xue et al., 2020), and whether to carry out age-matched donor transplantation without the interference of PD traditional drugs and the influence of dietary habits needs further evaluation. However, some practical problems need to be addressed including the management of fecal transplantation, removal of specific microorganisms, and influence of the optimal amount and frequency of transplantation (Kellermayer, 2019).

## Summary

In recent years, some breakthroughs have been made in the understanding of the intestinal origin of Parkinson’s disease. The mechanism may involve transfer of the Lewy body pathology through the brain-gut nerve axis, structural changes of intestinal flora to cause inflammation and oxidative stress in peripheral organs and brain, or the effect of a variety of mixed factors. However, the relationship between the intestinal tract and Parkinson’s disease is inseparable, which is supported by the non-motor symptoms and primarily gastrointestinal symptoms in early PD patients. The mechanism may be the imbalance of harmful intestinal flora, resulting in the increase of abnormal α - SYN in peripheral blood, which is then spread along the vagus nerve into the brain stem and finally to the brain. The retrograde movement mode of α - SYN is usually completed by the combination of vesicular exocytosis release mode and micro pinocytosis uptake mode besides the auxiliary exosome transmission mode. Currently, there are many clinical studies on intestinal flora in PD, but the characteristics of the flora are different in these studies, and it is difficult to infer the same specific flora of PD due to different research standards. In general, current clinical studies generally support that the abundance of pro-inflammatory and harmful bacteria increases, whereas the abundance of anti-inflammatory bacteria decreases. Although intestinal microbiological therapy for Parkinson’s disease is still in the initial stage with many practical problems to be solved, there are benefits in intestinal microbiological treatment of PD. Currently, there is still much room for the research of PD and intestinal microbiota. It is expected that, in the future, a clear relationship will be revealed between PD and intestinal microbiota, and active regulation of intestinal microbiota will be able to alleviate the clinical symptoms of PD patients.

## Author Contributions

Study design: Z-JC and X-FL. Data collection: Z-JC, C-YL, L-QY, S-MR, Y-MX, and LC. Data analysis: Z-JC, X-FL, and B-LG. Writing of the original version: Z-JC. Revision: B-LG. All authors contributed to the article and approved the submitted version.

## Conflict of Interest

The authors declare that the research was conducted in the absence of any commercial or financial relationships that could be construed as a potential conflict of interest.
